# Effectiveness of WHO mhGAP-IG in identification and management of depression by primary level non-specialist health workers – protocol of a cluster randomized controlled trial from India

**DOI:** 10.1186/s12889-026-28263-7

**Published:** 2026-07-02

**Authors:** Archana Siddaiah, Harshal Sathe, Uttara Chari, Madhu Mitha Manivannan, Roshan Rathod, Ashwini Kudlekar, Abhishek Urkude, Bharat Kalidindi, Dhinagaran Devadass, Rajini Parthasarathy, Rameshwar Paradkar, Sally John, Abhishek Raut, John Michael Raj, Triptish Bhatia, R. P. Beniwal, Samir Kumar Praharaj, Johnson Pradeep Ruben, Smita Deshpande

**Affiliations:** 1 Isaac Centre for Public Health, Indian Institute of Science, Bengaluru, India; 2 Department of Psychiatry, Mahatma Gandhi Institute of Medical Sciences, Warud, India; 3 Department of Psychiatry, St. John’s Medical College and Hospital, St. John’s National Academy of Health Sciences, Bengaluru, India; 4 St. John’s Research Institute, St. John’s National Academy of Health Sciences, Bengaluru, India; 5 Department of Health and Family Welfare, Government of Karnataka, Bengaluru, India; 6 Department of Health and Family Welfare, Government of Maharashtra, Mumbai, India; 7 Department of Biostatistics, St. John’s Medical College, St. John’s National Academy of Health Sciences, Bengaluru, India; 8 Department of Psychiatry, Atal Bihari Vajpayee Institute of Medical Sciences and Dr. Ram Manohar Lohia Hospital, New Delhi, India; 9 Department of Psychiatry, Kasturba Medical College, Manipal Academy of Higher Education, Manipal, Karnataka India

**Keywords:** Depression, Psychological intervention, Primary care, Mental healthcare, Non specialist health workers, Primary health workers, Trial, Mobile application, District mental health program, India.

## Abstract

**Background:**

Reports suggest that > 90% of the cases go undetected in low- and middle-income countries (LMICs) leading to a huge treatment gap. Evidence shows that task-sharing interventions such as training HWs, screening led to improved detection of depression at the primary level. In this regard, the World Health Organization (WHO) developed the mhGAP-IG to aid non-specialist HWs to detect and manage common mental health disorders such as depression to bridge the gap. The present study aims to design and test the effectiveness of a digital mental health intervention designed by contextualizing e-mhGAP in improving the detection and management of depression at the primary care level.

**Methods:**

Formative phase will involve qualitative interviews, literature review aimed at co-developing a mobile application with the district mental health program. This will be followed by a cRCT. The trial will be conducted in eight PHCs, located in Tumkur district, Karnataka, and Wardha district, Maharashtra. Randomisation will be done at the PHC level. We plan to enrol non-specialist HWs working in these PHCs and a sample of 2400 adults who are availing the PHC services. Intervention PHC will receive training for HWs and a mobile application. Control PHCs will follow usual care. Both the groups will be encouraged to identify, manage, and follow-up people with mild depression. Study tools include PHQ-2, PHQ-9, acceptability of intervention measure, and Eq. 5D5L instrument. Change in the detection rates of depression between intervention and control arms will be the primary outcome. Cost effectiveness analysis will be done.

**Discussion:**

This trial advocates for integrating mental health services into primary healthcare, which aligns with the Comprehensive Mental Health action plan that seeks to enhance the capacity of non-specialist HWs in mental healthcare delivery. Co-development with the district mental health program, features of app such as offline functionality, multimedia visuals, and follow-up tracking are some of the strengths. If this intervention is found effective, then it could significantly impact the treatment gap for depression by integrating mental health within the ambit of primary care model across India.

**Trial registration:**

Prospectively registered with the Clinical Trials Registry of India on 23/04/2024 (Reference number- CTRI/2024/04/066142).

**Supplementary Information:**

The online version contains supplementary material available at https://doi.org/10.1186/s12889-026-28263-7.

## Background

Globally 280 million people were suffering from depressive disorders, and it accounted for the largest proportion of mental disorder Disability Adjusted Life Years (DALYs) of 37.3% in 2019 [1]. In India, depression affected over 5% of the adult population [2]. Despite availability of effective interventions, the vast majority of people with depression do not receive any care [3, 4]. Untreated depression is a leading cause of disability and suicide [5]. Compared to general population, people with depression have a 40–60% higher chance of premature mortality [6–8]. Yet, detection rates of depression in the primary care settings is extremely low and reports suggest that > 90% of the cases go undetected in low- and middle-income countries (LMICs) leading to a huge treatment gap [9–10].

India provides health services including mental healthcare through a three-tier public health system [11]. Through the National Mental Health Program (NMHP), India envisages providing universal access to mental healthcare [12]. The District Mental Health Programme (DMHP) under NMHP focuses on delivering mental healthcare in a decentralized manner. In many states, DMHP has launched specific programs to integrate mental healthcare into public health facilities [13]. However, the success of these programs are limited to district level [14]. 

One of the barriers to integrating mental healthcare into primary care includes shortage of human resources [3, 6]. India faces hurdles in delivering mental healthcare due to a shortage and unequal distribution of trained mental health professionals between urban and rural areas [3]. Low mental health literacy, persisting stigma and discrimination in rural India, further exacerbates this issue [15]. Therefore, task-sharing, a process of training non-specialist health workers (HWs) to contribute to detecting, diagnosing, and treating various mental disorders, is a promising strategy [16]. Evidence shows that interventions such as training HWs, screening, feedback, and standard guidelines led to improved detection of depression at the primary level [17]. Increasingly, such task sharing strategies targeting primary-level HWs are being emphasized by the DMHP [14]. 

A review of psychosocial interventions for common mental disorders by non-specialist HWs in LMICs demonstrated the effectiveness of these interventions. Studies from India targeting non-specialist HCWs to manage mental disorders have been encouraging [18–21]. Some demonstrated the effectiveness of task-sharing interventions in the management of depression [22]. Hence, task-sharing is a promising approach for overcoming workforce shortages and strengthen detection and management of common mental disorders [16, 23]. 

The framework proposed to illustrate the role of technology in supporting HWs in delivering evidence-based mental healthcare includes competency-based training; digital interventions to support delivery of mental health care; and digital strategies to facilitate supervision and promote retention [23, 24]. However, training alone is insufficient in improving detection rates of depression [5]. Enhancing HWs’ training using validated digital tools for patient screening can be more effective. This framework helps in understanding the potential of digital interventions in maximizing skills of HWs in dealing with people with depression. Mobile health interventions have already shown potential in community settings—notably, the MITHRA application, which provided depression screening and brief interventions for women in rural India through self-help groups [25, 26]. 

The World Health Organization (WHO) developed the Mental Health Gap Action Programme Intervention Guide (mhGAP-IG), for primary-level non-specialist HWs wherein evidence-based algorithms aid HWs in managing mental health conditions, while the training packages and implementation tools facilitate integration with the health system [27]. mhGAP-IG has been implemented in over 100 countries [22, 28–30]. WHO released a smartphone-based e-mhGAP application in 2017 [31]. The e-mhGAP faced challenges like limited patient-specific interventions, offline functionality, and language support [32, 33]. While using mobile applications, diagnostic suggestions, follow-up reminders, offline access have been reported to be beneficial by HWs, but barriers included internet reliance, and distraction in the communication between patients and HWs [32]. A feasibility cluster randomized controlled trial (cRCT) reported increased usability of the application, higher satisfaction, and cost-effectiveness [31]. An Indian study found training completion rate for a digital program on depression to be satisfactory. However, challenges such as mobile network issues, difficulty navigating the content, and device hardware problems highlighted the need for augmented digital infrastructure [34]. 

### Rationale

There is a lack of systematic research on using e-mhGAP for managing depression in India [30]. The present study is designed to address challenges and limitations based on the evidence originating from LMICs. For instance, the mhGAP is only available in Marathi language [35]. For successful implementation, the intervention must be contextually adapted, available in regional languages and provide support for trainees [33, 36]. Therefore, this study aims to design a digital intervention by contextualizing e-mhGAP and test it in rural Karnataka and Maharashtra. Additionally, we seek to improve the psychological intervention of the depression module by incorporating multimedia to help HWs deliver mental healthcare confidently. The study’s goal is to integrate mental health services into primary care through a technology-driven approach which aligns with that of NMHP. In summary, task-sharing and the use of a novel mobile application in rural India hold promise in reducing the prevailing treatment gap for depression.

## Methods

### Objectives

Primary:

1. To co-develop a mobile application based on e-mhGAP IG for the detection and management of persons with mild depression by HWs.
2. To evaluate its effectiveness of this digital mental health intervention in the detection and management of persons with mild depression by HWs, compared to usual care in rural PHCs in two states of India.

Secondary:

3. To determine the acceptability and feasibility of this digital mental health intervention among HW.
4. To determine the cost-effectiveness of digital mental health intervention in improving the detection and management of mild depression by HWs.

### Study design

This will be an equal-arm parallel-group cRCT across two sites (Tumkur and Wardha). The cluster randomized design was chosen to ensure balance between methodological rigour and real-world feasibility focussed on conducting the research under practical primary care conditions relevant for programmatic decision making.

### Study setting

The trial will be conducted in eight PHCs, located in Tumkur district, Karnataka, and Wardha district, Maharashtra. Tumkur district has ten administrative sub-divisions and 150 PHCs, while Wardha district has eight sub-divisions and 33 PHCs. The PHCs are located between 30 and 100 km from the study sites. PHCs will be ranked based on the criteria given in Table 1. The top 20 ranked PHCs will be listed in descending order, and four PHCs will be randomly selected from each site.

**Table 1:** Criteria for selection of PHCs

PHC selection criteria	Ranking based on
*1. Distance from the study site to the PHC*	Farther less score
*2. Population catered by the PHC*	≥ 30,000 higher score
*3. Number of HWs working in the PHC*	≥ 10 higher score
*4. Monthly footfall of adult patients in the PHC*	≥ 2000 higher score
*5.Ongoing mental health research in the PHC*	Absent more score

### Study participants and eligibility criteria

Non-specialist HWs: As per the Indian Public Health Standards, non-specialist HWs at the level of PHC include staff nurses, auxiliary nurse midwives, community health officers, health assistants, and health educators [37]. HWs belonging to these groups will be recruited based on the inclusion criteria, which include ≥ 12 years of schooling and work experience of ≥ 1 year at the study PHC. Pregnant HWs will be excluded.

Adult participants: This includes adults who visit the study PHCs to avail health services. Those aged between 18 and 60 years, able to understand the local language, and who are likely to stay within the PHC area for the duration of the study will be eligible to participate. Persons with acute medical illness, pre-existing psychiatric conditions, and pregnant or lactating mothers will be excluded.

Stakeholders: Additionally, we will include DMHP administrators, psychiatrists, psychologists, psychiatric social workers, medical officers, technological experts, and HWs from non-study PHCs during the formative phase of the research.

### Interventions

**Fig. 1:**
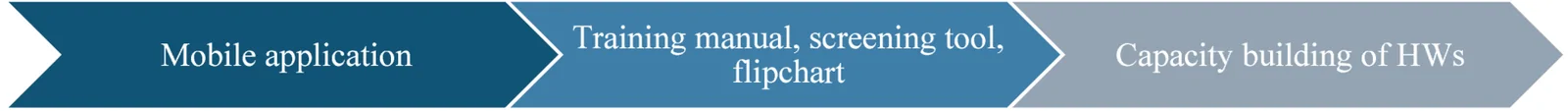
Study intervention package

The intervention includes capacity building for HWs, provision of a mobile application containing modules on screening and psychological intervention for managing mild depression (Fig. 1).

*Co-designing a mobile application*: The existing English e-mhGAP depression module will be culturally adapted to the study setting using a mixed-methods approach. A literature review and technical review of existing m-health applications used in various national health programs will be carried out. To understand the needs, facilitators, and barriers for implementing the digital mental health intervention, we will conduct in-depth interviews (IDI) and focus group discussions (FGDs). We plan to do 30 IDIs and 10 FGDs with all the stakeholders mentioned under study participants. Over the next six months, the research team will conduct 2–3 workshops with all stakeholders and application developers to co-design a mobile application containing modules on screening and managing depression through psychological interventions.

The co-developed application will include initial screening using Patient Health Questionnaire-2 (PHQ-2) [38] and assessment using Patient Health Questionnaire-9 (PHQ-9) [39] tools, psychological intervention modules, handling participant follow-ups, and referrals. We intend to improve the psychological intervention by plugging multi-media content that can aid HWs in counselling participants. One of the salient features will include offline functionality and multi-lingual option. Additionally, tutorials for HWs, user profile management, and action-oriented dashboards will also feature in the application. The software will be developed using the Agile methodology, employing iterative and incremental development cycles to ensure continuous refinement and user-centric improvements. The application will be designed for deployment on both web and Android platforms, utilizing modern open-source frameworks and tools. Development will adhere to the technical standards and guidelines set forth by the Ayushman Bharat Digital Mission (ABDM), ensuring interoperability, data security, and compliance with national digital health infrastructure norms.

Training toolkit for capacity building of HWs: Based the WHO mhGAP-IG [27], a context-specific training toolkit will be prepared which will have a training manual, screening and assessment algorithms, and flipcharts. A panel of mental health experts will face-validate the toolkit. Bilingual experts will translate the toolkit into the local languages (Kannada, Marathi, and Hindi) using a transcultural translation and adaptation (TTA) process [40, 41], which will be reviewed by the research team for accuracy. Back translation will be done to address any discrepancies and finalise the contents.

Capacity building of HWs: HWs in the intervention PHCs will undergo a three-day training led by a psychiatrist and research team using the principles of adult learning. Training topics are presented in Table 2. Short lectures, role-plays, case vignettes discussions, games, group activities, and video demonstrations will be used for training. Each HW will receive a training toolkit. Pre- and post-training assessments of HWs will be conducted using mhGAP knowledge questionnaire. HWs will then receive the mobile application for installing on their smartphone. To enhance HWs’ competencies required for counselling, training will be supplemented by HWs shadowing DMHP psychiatrists at the mental health camp. One refresher session will be held midway through the trial.

**Table 2:** Contents of training for capacity building of HWs

Training day	Training topics
*Day 1 (4.5 h)*	Pre-training assessment
	Introduction to common mental health issues Integrating mental health into primary care
	Essentials of mental health care and clinical practice: assessments, management, & practice
	Introduction to depression
	Absent more score
*Day 2 (4.5 h)*	Assessment for (mild/moderate/severe) depression
	Management of depression and non-pharmacological interventions
	Introduction to e-mhGAP and Screening using e-mhGAP
*Day 3 (4.5 h)*	Psychoeducation using e-mhGAP
	Follow-up using e-mhGAP
	Reporting and referrals, assessing suicidal tendency
	Post training assessment and wrap up

### Pilot phase

To assess the feasibility, recruitment rate, and logistical requirements, a pilot will be conducted in two urban PHCs. We plan to include 10–20 HWs working in these PHCs as per the eligibility. HWs at one PHC will receive the intervention package, while HWs at the other PHC will receive a brief sensitisation on mental health issues. The pilot will be conducted over three-months, during which HWs at the intervention PHC will screen 25–50 adults for depression and manage using the mobile application. HWs at the control PHC will screen 25–50 adults for depression and manage with usual care. HWs will counsel participants screened positive for mild depression at the time of identification, and during the 3rd -4th, 7th-8th, and 11th-12th weeks. Recruitment rate, intervention fidelity, and drop-out rate will be measured to guide the main trial protocol.

### Allocation and sequence generation

Randomisation will be done at the PHC level, separately for Tumkur and Wardha districts. Allocation sequence will be independently generated by the study biostatistician. Eight PHCs will be included in the cRCT, with 1:1 allocation ratio.

### Implementation

**Fig. 2:**
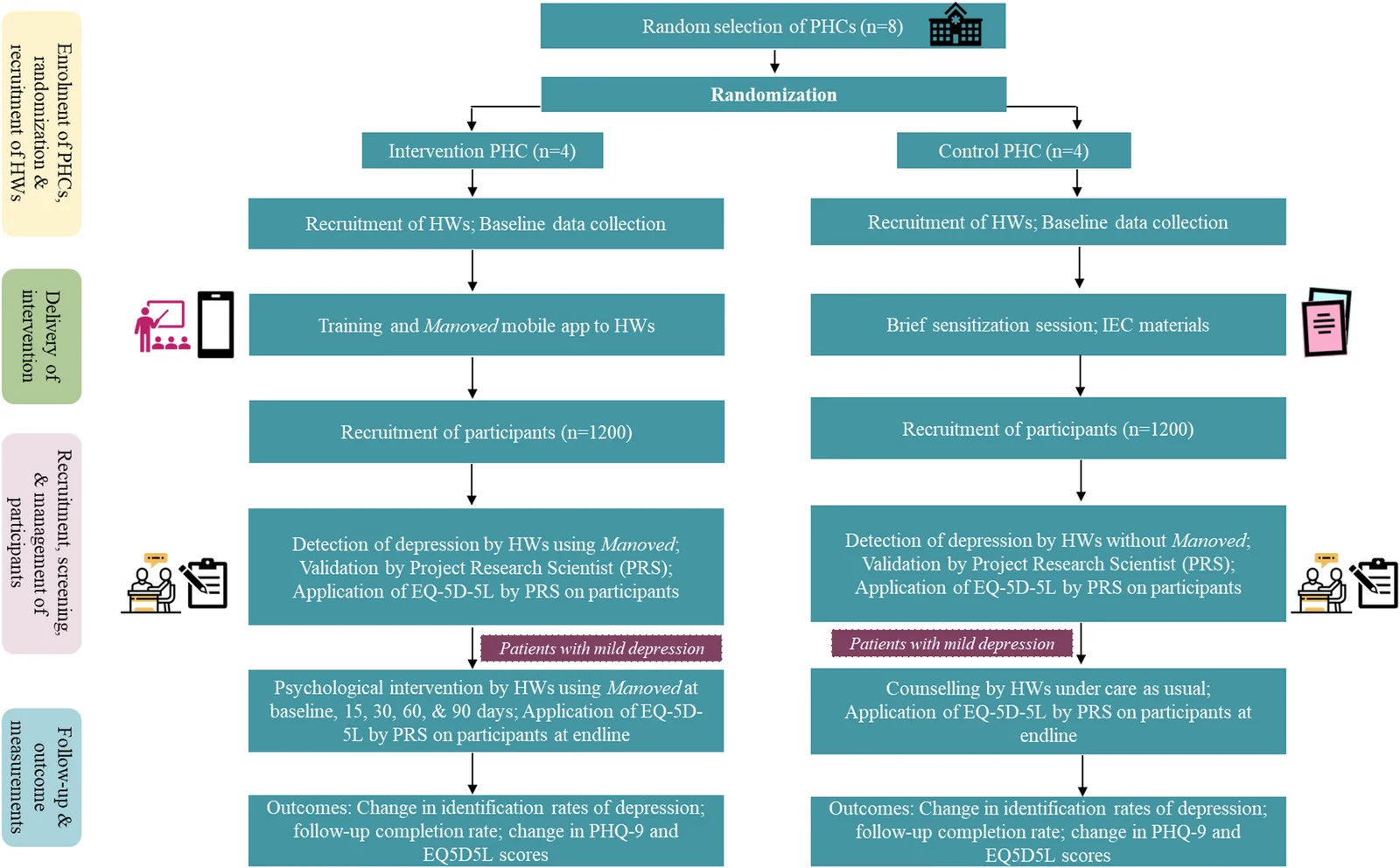
Consort flow diagram

The project research scientist (PRS) will enrol the study participants after due informed consent. The cRCT will be conducted in four different phases (Fig. 2).

Phase I: In this, baseline data collection using outpatient records in all study PHCs will be carried out to determine the PHC footfall, number of adults diagnosed, managed, or referred for mental health conditions over the past six months.

Phase II: PHCs will be randomised and allocated into the intervention arm (*n* = 4) and the control arm (*n* = 4). Subsequently, intervention package will be delivered to HWs in the intervention PHCs. HWs at the control PHCs will receive a one-day sensitisation session on mental health conditions, including brief psychoeducational counselling on depression.

Phase III: Screening, assessment, psychological counselling of adult participants by HWs, and referrals will take place during this phase. In both intervention and control PHCs, adults attending the study PHCs will be enrolled consecutively ss per the eligibility criteria. They will be allotted to each HW depending on the availability of HW in the PHCs for screening, assessment, and management of depression.

Phase IV: In both arms, participants identified with mild depression will receive psychological counselling from HWs and will be followed up as outlined in the pilot phase. Each follow-up counselling session will last 10 min per participant. PRS will give telephonic reminders to participants to reduce dropouts. Screening, assessment, and management of mild depression will be done using application in intervention PHCs and through usual care in control PHCs by the HW.

Validation by PRS: At the time of baseline assessment, the PRS will validate all participants identified with mild depression by HWs. Along with this, PRS will also randomly conduct a validation check PHQ 9 scores in participants identified as normal by HWs. To document changes in PHQ-9 scores, the PRS will administer the PHQ-9 to participants at all follow-up visits. PRS will do a health-related quality of life (HRQoL) assessment at baseline and the last follow-up.

Referral: Participants with PHQ-9 scores ≥ 10 or having suicidal ideation will be referred to the DMHP psychiatrist at the sub-district hospital. The PRS will contact participants telephonically to ensure they receive appropriate care. Throughout the study, the research team will provide weekly supportive supervision to all HWs in both arms to provide feedback and address any issues they may face.

Reimbursements: HWs and adult participants will receive a nominal monetary incentive as reimbursement for their time spent towards the study. The amount will be set at a level that ensures it does not interfere with the voluntariness of participants’ consent.

### Blinding

Due to the nature of the intervention, blinding will not be feasible at the level of HWs. Measures will be taken to minimise ascertainment bias by training the research team in standardised data collection methods. The investigators will monitor this.

### Outcome assessment

Outcomes will be measured at both the PHC and participant levels and have been outlined in Table 3.

**Table 3:** Study outcome according to various phases and data sources

Outcome measures	Respondents	Data collection type
*Objective 1*		
* 1. Themes and sub-themes*	DMHP stakeholders, HWs, community members	Qualitative interviews
* 2. Pre and post training scores*	HWs	Study questionnaire
* 3. User validity testing*	DMHP stakeholders, HWs	Observation checklist
*Objective 2*		
* 1. Detection rates of depression*	HWs, adults	Study monitoring forms; application data
* 2. Treatment completion rates*	HWs, adults	Study monitoring forms; application data
* 3. Referral rates*	HWs, adults	Study monitoring forms; application data
* 4. HRQoL scores*	Adults	Study questionnaire
*Objective 3*		
* 1. Acceptability*	HWs	AIM; Qualitative interviews
* 2. Feasibility*	HWs	AIM; Qualitative interviews
* 3. Fidelity*	HWs	AIM; Qualitative interviews, Study monitoring forms
* 4. Theme and sub-themes*	HWs	Qualitative interviews
*Objective 4*		
* 1. Incremental cost effectiveness ratio*	HWs, adults, DMHP stakeholders	Study questionnaire, administrative data, project reports

Acceptability and feasibility: This will be assessed using both quantitative and qualitative methods in the intervention arm. (1) Feasibility: Recruitment and attrition rates will be estimated, and intervention fidelity will be monitored using standard study forms, with any protocol deviations documented. (2) Acceptability: HWs’ acceptance of the intervention will be measured using the Acceptability of Intervention Measure (AIM) instrument. Developed by implementation scientists, the AIM instrument has displayed good psychometric properties [42]. At the end of the trial, FGDs will be conducted with the HWs to understand their perceptions and experiences of using the application.

Cost-effectiveness of the intervention: Cost-effectiveness analysis will be conducted from a societal perspective by listing, measuring, and valuing all costs involved in delivering the intervention. An ingredients approach will be used to collect information on quantities and prices of all resources across three categories: (a) Costs of providing the intervention—includes direct and indirect costs training, application development, consumables, time, human resource, outpatient visits, and counselling. (b) Costs of accessing the intervention—includes patient resources such as travel costs to the PHC and opportunity costs. (c) Overhead costs—includes personnel costs based on salaries and time spent on the intervention as well as utilities. Cost valuation will ensure transferability of cost over time, with the Consumer Price Index used to adjust for inflation. A 3% discount rate, as recommended by the WHO, will be applied.

### Participant timeline

The participant timeline has been presented in Table 4.

**Table 4:** Participant timeline

	Year 1				Year 2				Year 3			
Project activities	1	2	3	4	1	2	3	4	1	2	3	4
Administrative permissions	X											
Development of mobile application		X	X	X								
Development of training modules			X	X								
Pre-trial pilot phase												
Pilot study				X	X							
Trial phase												
Selection of PHCs					X							
Recruitment of HWs					X							
Baseline data collection					X	X						
Randomisation						X						
Delivery of intervention						X	X	X				
Recruitment of adult participants							X					
Screening and counselling by HWs							X	X	X			
Follow-ups and outcome assessments							X	X	X	X		
Post-trail phase												
Measurement of costs							X	X	X	X		
Acceptability & feasibility measures										X		
Analysis, & report preparation									X	X	X	X
Project conclusion												X

### Sample size

The sample size was calculated by considering a 5% detection rate of depression at primary care level [9, 10] and assuming a difference in detection rate by 20% between the intervention and control arm. Assuming an equal cluster size of 300, we will require 4 PHCs in each arm. Considering 20% attrition rate, intra-class correlation (ICC) as 0.1 [43], with 80% power, 5% alpha error for two-sided test, the minimum sample size required is 1200 adults per arm. Assuming the usual recovery rate for depression at the primary level to be 54% and an expected difference of 20% in the recovery rates between intervention and control arms, with 80% power, 5% alpha error for two-sided test, the required estimated sample size is 89 patients in each arm. Therefore, a minimum of 1200 participants per arm will be sufficient.

### Recruitment

Recruitment will take place in the PHCs. Medical Officers will be requested to direct adults attending the outpatient department to the PRS stationed at these PHCs. PRS will check eligibility before obtaining written informed consent and contact details for enrolment. We will target 70–80 participants per month per PHC to recruit 300 participants from each PHC. Awareness campaigns will be carried by distributing study brochures, posters within the PHC, conducting community meetings to achieve desired recruitment.

### Data collection methods

Qualitative interviews will be conducted using IDI and FGD guides, prepared by the research team. Pre-training and post-training knowledge of HWs will be assessed using mhGAP knowledge questionnaire [44]. 

Screening and assessment of participants will be done using the PHQ-2 and PHQ-9. PHQ-2 has been recommended as a pre-screen prior to administering PHQ-9 items [45]. A score of ≥ 3 requires further assessment with PHQ-9 to determine severity of depression. The PHQ-9, designed for primary care settings, consists of 9 items which measure the frequency of depressive symptoms over the past fortnight. A multicentric Indian study reported PHQ-9 reliable for screening depression by HWs [46]. Table 5 provides the operational definitions of our study.Acceptability and feasibility of the intervention will be assessed using AIM instrument [47]. 

The HRQoL will be measured at baseline and endline by the PRS using EUROQUAL instrument (EQ-5D-5 L), the norms of which are available for the Indian population [48]. 

Detailed costing of every single ingredient will be gathered for doing cost-effectiveness analysis.

**Table 5:** Operational definition for PHQ-9 used in the study

Operational definition	PHQ-9 score
*1. Normal*	< 5
*2. Mild depression*	5–9
*3. Moderate depression*	≥ 10
*4. Severe depression*	≥ 15
*5. Recovery*	< 5

The socio-demographic details from participants will be used to generate a unique identification number (UID).

In the intervention arm, screening, assessment, and counselling of participants by HWs, validation by the PRS and follow-up data will be captured in the mobile application. This data will get stored in the application backend which can be downloaded in Microsoft Excel format. In the control arm, screening, assessment, follow-up, and validation data will be captured using a case record form (CRF). This along with the HWs knowledge scores, EQ-5D-5 L scores, AIM scores, and costing data will be collected using Epicollect5 with appropriate quality checks to minimise errors. Respective site PIs will constantly monitor all the data captured through the application and by Epicollect5.

### Plans to promote participant retention and complete follow-up

Strategies to minimise loss to follow-up will include using simplified application usage to reduce the burden on HWs, flexibility in scheduling follow-ups, and periodic telephonic reminders to adult participants. Regular weekly meetings with the HWs will be held for supportive supervision and to address their concerns. Details of drop-outs or participants lost to follow-up will be documented appropriately.

### Ethical data management

Research team will be trained in collecting data under strict confidentiality, using standard study tools. Consent forms and CRF will be appropriately stored, ensuring protection from damage, fire accidents, or theft by locking them in a designated, secure office. All data with demographic details will be delinked from the indirect identifiers. Before delinking, the master sheet that links the participant’s name and phone numbers linked to their UID will be securely stored in a password-protected computer accessible only to PIs. A Data and Safety Monitoring Board (DSMB) will be constituted to monitor and audit the conduct of the research.

### Statistical methods

The qualitative data will be audio-recorded and transcribed verbatim. The transcriptions and the field notes from IDIs and FGDs will be manually coded. An inductive-deductive approach will be used to identify key themes and sub-themes. Reporting will be done using the Consolidated Criteria for Reporting Qualitative Studies (COREQ) checklist [49]. 

After checking for normality, continuous data will be expressed using mean (standard deviation) or median (interquartile range). Categorical data will be described using frequency and percentages. The difference in the detection and recovery rates between intervention and control arms, as well as associated risk factors for depression, will be compared using generalised estimating equation (GEE) analysis with a log link and exchangeable correlation structure to adjust for cluster effects. The change in EQ-5D-5 L scores during the follow-up will be compared between two groups using a mixed linear model. A p-value less than 0.05 will be considered significant. Missing data will be handled using multiple imputation methods.

Standard health economic methods will be applied to determine the incremental cost-effectiveness ratio (ICER) based on the costs and outcomes associated with the intervention. Sensitivity analyses will be conducted to test the robustness of the results. All analyses will be carried out using R software. Tree age software will be used to perform cost-effectiveness analyses.

### Data monitoring

Both the site PIs will ensure the completeness of the data by continuous monitoring. DSMB will be established to oversee the conduct of the research and will consist of three independent reviewers who are experienced in conducting cRCT and will be common across the two sites. DSMB will review all procedures related to enrolment, protocol deviations, protection of study participants, confidentiality procedures, and adverse events. DSMB will also review the consent forms collection, storage, and processing of the participant data.

### Harms

The intervention is anticipated to pose minimal risk. Research staff will be trained to identify, assess, and report adverse events according to pre-established protocols. In case of an adverse event, such as a breach of confidentiality or high levels of distress, the research team will assess such concern by meeting with the participant. Participants requiring psychological support will be referred to our liaison DMHP Psychiatrist. The PRS will follow up with them to ensure their distress has been stabilised. DSMB will be provided with reports of all adverse events before their meetings and of serious adverse events within 5–7 days.

## Discussion

### Implication of the trial

This trial advocates for integrating mental health services into primary healthcare, which aligns with policies of NMHP and the Comprehensive Mental Health action plan that seeks to enhance the capacity of non-specialist HWs in mental healthcare delivery [2, 7]. The cRCT focuses on task-sharing, a well-documented strategy, proven effective in LMICs, for improving mental health service delivery [3]. The trial aims to adapt the mhGAP-IG depression module by involving diverse stakeholders such as the DMHP psychiatrists, psychologists, psychiatry social workers, public health experts, medical officers, and HWs. Capacity building of HWs based on the mhGAP-IG, offline functionality of the mobile application, and language flexibility are some of the strengths. Challenges like poor retention of knowledge and limited confidence of HWs will be addressed through the provision of in-app tutorials designed for on-demand learning, as well as continued supportive supervision and feedback.

One of the trial highlights include the co-development of a digital mental health intervention involving various system-level stakeholders. We hope that the co-development process will increase acceptance of our intervention within the health system. Evidence shows that involving multiple stakeholders improved the feasibility of implementing the mhGAP modules [50, 51]. The application can facilitate easy tracking of patient progress and enable referrals to specialists. Availability of the intervention in local languages will ensure that HWs can use the guidelines seamlessly resulting in increased patient engagement.

It is hoped that the intervention will improve HW’s competence in managing other priority mental health conditions in the long run. Findings from this cRCT, such as the cost-effectiveness of the digital mental intervention, could influence policy decisions leading to its integration into India’s primary healthcare framework. This aligns with India’s digital health mission, where applications like this can strengthen the existing digital health ecosystem. A growing focus on mental health may reduce stigma in the community and enhance health seeking behaviour.

### Anticipated challenges

Factors such as low digital literacy among HWs, poor internet connectivity, and distrust of digital tools may hinder HWs from effectively using these tools, adapting to novel technologies, or resolving technical issues without assistance. Additionally, power outages in rural areas pose challenges to seamless integration of digital solutions into HW’s routines. While the application’s offline functionality helps address some of these issues, challenges like delayed updates, limited device storage, and lack of real-time support may persist. Adding mental health screening and management to their already substantial workload could lead to resistance, as HWs may perceive it as an additional burden without adequate time or resources. Lastly, improving HW’s competencies in delivering psychological interventions within a short time could also be a challenge.

## Conclusions

The proposed trial aims to address the increased treatment gap for depression in rural India through task-sharing using digital mental health interventions. Adapting mhGAP-IG into Kannada, Marathi, and Hindi enhances accessibility and cultural relevance of our intervention. Key strengths include integrating evidence-based digital mental health tools at the primary level. However, challenges such as technological barriers, workforce burden, stigma, and sustainability must be addressed for successful implementation. Ensuring adequate training, providing ongoing support, and securing policy backing are crucial for success. If implemented effectively, this intervention can make mental health services more inclusive and accessible in rural India.

## Supplementary Information

Supplementary Material 1.

## Data Availability

No datasets were generated or analysed during the current study.

## Abbreviations

AIM: Acceptability of intervention measure
COREQ: Consolidated criteria for reporting qualitative studies
cRCT: Cluster randomised controlled trial
CRF: Case record form
CTRI: Clinical trials registry of India
DMHP: District mental health programme
DSMB: Data and Safety Monitoring Board
EQ5D5L: EuroQol–5 dimensions–5 levels
FGD: Focus group discussions
GEE: Generalised estimating equation
HRQoL: Health–related quality of life
HWs: Health workers
ICER: Incremental cost effectiveness ratio
IEC: Institutional ethics committee
ICC: Intra–class correlation
IDI: In–depth interviews
LMICs: Low middle income countries
mhGAP: IG–Mental health gap action programme intervention guide
NMHP: National mental health program
PHC: Primary healthcare centre
PIs: Principal investigators
PRS: Project research scientist
PHQ: Patient health questionnaire
TTA: Transcultural, translation and adaptation
UID: Unique identification number
WHO: World Health Organization
